# Invasive versus noninvasive measurement of allergic and cholinergic airway responsiveness in mice

**DOI:** 10.1186/1465-9921-6-139

**Published:** 2005-11-25

**Authors:** Thomas Glaab, Michaela Ziegert, Ralf Baelder, Regina Korolewitz, Armin Braun, Jens M Hohlfeld, Wayne Mitzner, Norbert Krug, Heinz G Hoymann

**Affiliations:** 1 Fraunhofer Institute of Toxicology and Experimental Medicine (ITEM), Nikolai-Fuchs Str.1, 30625 Hannover, Germany; 2 Hannover Medical School, Department of Respiratory Medicine, Carl-Neuberg Str.1, 30625 Hannover, Germany; 3 Division of Physiology, Bloomberg School of Public Health, Johns Hopkins University, Baltimore, Maryland 21205, USA

## Abstract

**Background:**

This study seeks to compare the ability of repeatable invasive and noninvasive lung function methods to assess allergen-specific and cholinergic airway responsiveness (AR) in intact, spontaneously breathing BALB/c mice.

**Methods:**

Using noninvasive head-out body plethysmography and the decrease in tidal midexpiratory flow (EF_50_), we determined early AR (EAR) to inhaled Aspergillus fumigatus antigens in conscious mice. These measurements were paralleled by invasive determination of pulmonary conductance (GL), dynamic compliance (Cdyn) and EF_50 _in another group of anesthetized, orotracheally intubated mice.

**Results:**

With both methods, allergic mice, sensitized and boosted with A. fumigatus, elicited allergen-specific EAR to A. fumigatus (p < 0.05 versus controls). Dose-response studies to aerosolized methacholine (MCh) were performed in the same animals 48 h later, showing that allergic mice relative to controls were distinctly more responsive (p < 0.05) and revealed acute airway inflammation as evidenced from increased eosinophils and lymphocytes in bronchoalveolar lavage.

**Conclusion:**

We conclude that invasive and noninvasive pulmonary function tests are capable of detecting both allergen-specific and cholinergic AR in intact, allergic mice. The invasive determination of GL and Cdyn is superior in sensitivity, whereas the noninvasive EF_50 _method is particularly appropriate for quick and repeatable screening of respiratory function in large numbers of conscious mice.

## Background

Asthma is a complex disease associated with reversible airway obstruction of variable degree, airway inflammation, airway hyperresponsiveness (AHR) and airway remodeling. These hallmarks of asthma are being examined in murine models, with the goal of understanding the basic cellular and genetic mechanisms of allergic inflammation that underlie the immunologic basis of the disease [1]. To investigate the functional consequences of in vitro findings in the lung in vivo, determination of pulmonary function is an essential tool. Existing methods for measuring respiratory function in mice in vivo include invasive and noninvasive approaches [2,3]. The invasive recording of pulmonary resistance (RL) or pulmonary conductance (1/RL), and dynamic compliance (Cdyn) is the gold standard for precise and specific determinations of pulmonary mechanics [2,3]. Limitations of traditional invasive methodologies commonly involve surgical tracheostomy, anesthesia, and mechanical ventilation, all of which are procedures that may generate significant artifacts [2]. In addition, when tracheostomy is done, this method is limited to single-point measurements only, usually precluding the possibility of performing follow-up studies. A novel modification to this invasive technology has enabled repetitive invasive recordings of pulmonary mechanics in conjunction with local aerosol delivery in anesthetized, orotracheally intubated, spontaneously breathing mice [4].

Noninvasive determination of respiratory parameters in conscious mice is a convenient, repeatable approach for screening respiratory function in large numbers of animals. Here, the application of the empiric variable enhanced pause (Penh) has gained widespread popularity. A recent correspondence written by leading experts [5] has emphasized the danger of the increasing uncritical use of Penh, with potentially misleading assessment of pulmonary function in animal models of lung disease. Although noninvasive measurement of murine respiratory function has virtually become synonymous with the recently questioned Penh method [5-9], a variety of other noninvasive methods have been established [10-12]. We and others have described the utility of midexpiratory flow, as measured by head-out body plethysmography, as a physiologically meaningful, noninvasive parameter of bronchoconstriction for mice and rats [13-17]. No report has as yet directly investigated the ability and utility of repetitive invasive and noninvasive lung function methods to assess allergen-specific EAR and cholinergic airway hyperresponsiveness (AHR) in intact mice. The primary objective of this study in a mouse model of fungal asthma was to compare the capability of noninvasive EF_50 _measurements to reflect the allergen-specific and cholinergic AR as observed with invasive determination of pulmonary mechanics. Moreover, to support the argument that noninvasive EF_50 _measurement is more valid than Penh we sought to examine whether EF_50_, unlike Penh [18], parallels the actual changes in pulmonary mechanics in response to hyperoxia in C57BL/6 mice. Our results showed that, while the noninvasive measurement of EF_50 _presented greater variability than the classical invasive measurements of RL and Cdyn, the correlation was sufficiently strong to support the use of such noninvasive testing in repetitive measurements in invividual mice.

## Methods

### Animals and sensitization protocol

Pathogen-free, female BALB/c mice, 12–14 weeks of age, and female C57BL/6 mice (used only for hyperoxia exposures), 7–8 weeks of age (Charles River, Sulzfeld, Germany), were kept in a pathogen-free rodent facility and were provided food and water ad libitum. All animal experiments conformed to NIH guidelines and were approved by the appropriate governmental authority (Bezirksregierung Niedersachsen, Germany). Allergic BALB/C mice (n = 8) received an intraperitoneal and subcutaneous injection of soluble A. fumigatus antigens (5 μg each, Greer Laboratories Inc, Lenoir, NC, USA), dissolved in incomplete Freund's adjuvant in a volume of 0.1 ml given on day 0 and were boosted noninvasively by inhalation over 10 min in a closed chamber with 1 % of A. fumigatus aerosol dissolved in saline on day 14 (jet nebulizer, LC Star, 2.8 μm mass median aerodynamic diameter (MMAD), Pari GmbH, Starnberg, Germany).

On day 21, allergic mice were challenged once with aerosolized A. fumigatus followed by methacholine (MCh, Sigma, Deisenhofen, Germany) dose-response exposure 48 h later (d 23). The control group (n = 8) received the same treatment schedule but was boosted and challenged with saline before MCh exposure. This protocol was chosen to maximize the difference between allergic and control groups. For the noninvasive measurement of pulmonary function separate groups of A. fumigatus-sensitized and control mice were used (n = 8 each group).

### Noninvasive measurement of pulmonary function in conscious mice

Noninvasive respiratory function was assessed with a glass-made head-out body plethysmograph system for four mice as previously described [14,17,19]. Briefly, mice were placed in the body plethysmographs while the head of each animal protruded through a neck collar (9 mm ID, dental latex dam, Roeko, Langenau, Germany) into a ventilated head exposure chamber. Monitoring of respiratory function was started when animals and individual measurements settled down to a stable level. For airflow measurement, a calibrated pneumotachograph (capillary tube PTM 378/1.2, HSE-Harvard, March-Hugstetten, Germany) and a differential pressure transducer (Validyne DP 45-14, range ± 2 cm H_2_O, HSE-Harvard) coupled to an amplifier were attached to the top port of each plethysmograph. For each animal the amplified analog signal from the pressure transducer was digitized via an analog-to-digital converter (DT 302, Data Translation, Marlboro, MA). The pneumotachograph tidal flow signal was integrated with time to obtain tidal volume (VT). From these signals the parameters tidal midexpiratory flow (EF_50_), time of expiration (TE), tidal volume (VT) and respiratory rate (f) were calculated for each breath and were averaged in 5 s segments with a commercial software (HEM 3.4, Notocord, Paris, France).

During airway constriction the main changes in the tidal flow signal occur during the midexpiratory phase. We defined EF_50 _(ml/s) as the tidal flow at the midpoint (50 %) of expiratory tidal volume, and we used this as a measure of bronchoconstriction [12,14,17]. A reduction in EF_50 _of more than 1.5 Standard deviation (SD) of mean baseline value (which translates to a reduction of more than 20% versus baseline) is considered to indicate airway constriction. The degree of bronchoconstriction to inhalation challenge was determined from minimum values of EF_50 _and was expressed as percent changes from corresponding baseline values.

### Invasive measurement of pulmonary function

AR was assessed as an increase in RL or decreases in Cdyn and EF_50 _in response to aerosolized A. fumigatus or MCh in anesthetized, spontaneously breathing mice as previously described in detail [4]. Briefly, mice were anesthetized with intraperitoneal injections of metomidate (total dose: 38–60 mg/kg) and fentanyl (total dose: 0.02 – 0.06 mg/kg) with minimal supplementations as required. When an appropriate depth of anesthesia was achieved, mice were suspended by their upper incisors from a rubber band on a Plexiglas support. The trachea was transilluminated below the vocal cords by a halogen light source and a standard 20G × 32 mm Abbocath^®^-T cannula (Abbott, Sligo, Ireland) was gently inserted into the tracheal opening. The intubated, spontaneously breathing animal was then placed in supine position in a thermostat-controlled whole-body plethysmograph (type 871, HSE-Harvard, designed in cooperation with Fraunhofer ITEM). The orotracheal tube was directly attached to a pneumotachograph (capillary tube PTM T16375, HSE-Harvard) installed in the front part of the chamber. Tidal flow was determined by the pneumotachograph connected to a differential pressure transducer (Validyne DP 45-14, HSE-Harvard). To measure transpulmonary pressure (PTP) a water-filled PE-90 tubing was inserted into the esophagus to the level of the midthorax and coupled to a pressure transducer (model P75, HSE-Harvard). The amplified analog signals from the pressure transducers were digitized as described above for noninvasive measurements.

Pulmonary resistance (RL) and dynamic compliance (Cdyn) were calculated over a complete respiratory cycle using an integration method over flows, volumes and pressures as previously described [4,20]. The resistance of the orotracheal tube (0.63 cm H_2_O·s·ml^-1^) was subtracted from all RL measurements. RL, Cdyn, EF_50 _together with other basic respiratory parameters were continuously recorded with a commercial software (HEM 3.4, Notocord). For easier comparison of trends among all variables, RL was expressed as pulmonary conductance GL (GL = 1/RL).

Respiratory parameters were averaged in 5 s segments and minimum GL, Cdyn and EF_50 _values were taken and expressed as percent changes from corresponding baseline values. After the measurements on day 21, mice were removed from the chamber and extubated as soon as they began recovering from anesthesia.

### Administration of aerosols

After recording of baseline values, airway responsiveness (AR) to A. fumigatus 2 % or saline (control group) was determined in separate groups of conscious and intubated mice on day 21. On day 23, dose-response studies to aerosolized MCh were performed in the same mice.

For intubated mice, dried aerosols of A. fumigatus 2 % (inhaled dose: 8 μg) and MCh 5 % (inhaled doses: 0.05–2.5 μg) were generated by a computer-controlled, jet-driven aerosol generator system (Bronchy III, particle size 2.5 μm MMAD, Fraunhofer ITEM, licensed by Buxco, Troy, NY) as previously described (15, 21).

Conscious mice placed in the head-out body plethysmographs were exposed noninvasively to A. fumigatus (2 %, inhaled dose 32 μg) and MCh aerosols (0.5–3 %, cumulative inhaled doses: 3–14 μg) delivered by a Pari jet nebulizer as previously described [13,14,22]. In both systems, aerosol concentrations were determined by a gravimetrically calibrated photometer. The total inhalation doses of A. fumigatus and MCh were calculated based on the continuously measured aerosol concentrations and respiratory volume per min [4,21]. The results of the bronchoconstrictor response to MCh were expressed as PD50 which is the dose of MCh required to reduce either GL, Cdyn or EF_50 _to 50 % of their respective baseline values and was calculated from the dose-response curves.

### Exposure to oxygen

C57BL/6 mice were randomly assigned to two groups: The mice in the control group (n = 8 each) were kept in room air whereas the other group of 8 mice was exposed to 100 % oxygen for 48 h. Exposure to 100 % oxygen was performed in a sealed (25 L) Plexiglas chamber with a flow of 2 L/min as similarly described earlier [18]. The CO_2 _level in the chamber was maintained at 1 % by using a CO_2 _absorber (Drägersorb 800 plus, Dräger, Lübeck, Germany). Food and water were provided ad libitum.

### Bronchoalveolar lavage (BAL) cell counts

At the end of this protocol, total and differential cell counts from BAL samples using 2 × 0.8 ml aliquots of saline were determined as previously described (14), except that, recovery of BAL fluids was performed from the distal trachea in intubated animals.

### Statistics

Comparisons of baseline values between groups and intraindividual comparisons were analyzed by the Student's two-sided t-test, allergic responses of the group of allergic mice versus control mice were analyzed by one-sided t-test. P values < 0.05 were considered significant. Descriptive results were expressed as means ± SE unless indicated otherwise. Comparison of a new measurement technique with an established one is needed to see whether they agree sufficiently. A plot of the difference against the standard measurements will often appear to show a relation between difference and magnitude when there is none. A plot of the difference against the average of the standard and new measurements is unlikely to mislead in this way. Accordingly, the agreement between the invasive and noninvasive lung function methods was analyzed by the method of Bland and Altman [23]. Graphically, the difference of each pair of measurement was plotted against their mean values. Agreement was expressed as the mean differences over all measurements and their corresponding 95% confidence intervals (95% CI). The limits of agreement were expressed as the mean differences ± 2 SD of the differences, together with their 95% confidence intervals (95% CI). Statistics was performed with SPSS 11.5.

## Results

### Baseline values for respiratory parameters in conscious and anesthetized mice

To illustrate the impact of anesthesia on respiratory function, baseline respiratory parameters were measured in anesthetized and conscious mice. Table 1 presents the baseline values of respiratory parameters obtained from conscious and anesthetized BALB/c mice. There were significant differences in f, TE and EF_50 _values between anesthetized and conscious animals at baseline. In addition, no differences in respiratory parameters were observed between allergic and control mice at baseline when separated into conscious and anesthetized groups.

**Table 1:** Baseline values for respiratory parameters from allergic and control BALB/c mice

Respiratory parameters	Definition	Control mice conscious	Allergic mice conscious	Control mice anesthetized	Allergic mice anesthetized
VT, ml	tidal volume	0.21 ± 0.05	0.19 ± 0.04	0.14 ± 0.02	0.13 ± 0.02
f, breaths/min	respiratory frequency	198 ± 41	220 ± 23	129 ± 20*	124 ± 29*
TE, s	time of expiration	0.17 ± 0.06	0.14 ± 0.02	0.3 ± 0.04*	0.3 ± 0.05*
EF_50_, ml/s	tidal midexpiratory flow	2.05 ± 0.89	2.26 ± 0.46	0.93 ± 0.14*	1.12 ± 0.43*
GL, ml·s^-1^·cmH_2_O^-1^	pulmonary conductance	-	-	1.05 ± 0.36	1.29 ± 0.69
Cdyn, ml·cmH_2_O^-1^	dynamic compliance	-	-	0.037 ± .007	0.030 ± .008

Baseline values are means ± SD obtained from 8 animals per group during a 5 min control period from conscious and anesthetized, orotracheally intubated BALB/c mice. In comparison with conscious mice, EF_50_, TE and f values were significantly altered in anesthetized mice. No difference was found between allergic animals and control groups when separated into conscious and anesthetized mice. *P < 0.05 versus conscious mice.

### Comparison of invasive and noninvasive lung function measurements of EAR

**Figure 1:**
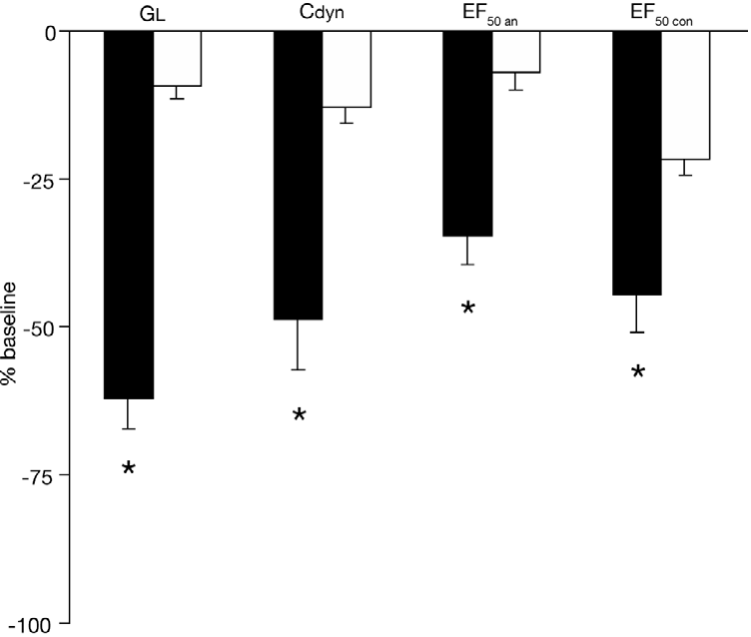
**Early airway responsiveness. **Invasive vs. noninvasive assessment of early airway responsiveness (EAR) to aerosolized Aspergillus fumigatus 2 %. Allergic (black columns) and control mice (white columns) were separated into groups of invasively and noninvasively monitored animals. The allergic mice showed significant reductions in simultaneously measured GL, Cdyn and EF_50an _(an: anesthetized), compared with control animals. Noninvasive determination of EF_50con _(con: conscious) elicited significant decreases in EF_50 _to inhaled A. fumigatus compared with control animals. EAR was expressed as % change from corresponding baseline values, which were taken as 0 %. Values are means ± SE, n = 8 per group, *p < 0.01 vs. control.

**Figure 2:**
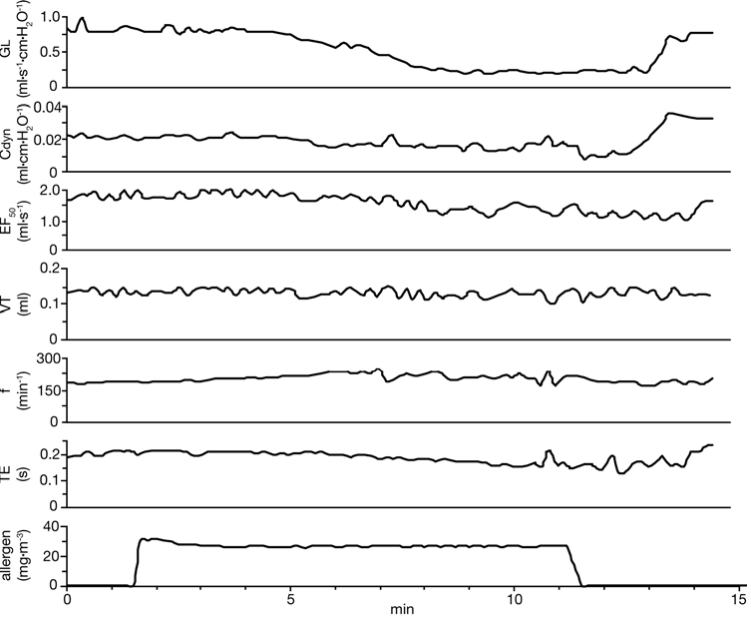
**Example of EAR. **Example of an early airway response (EAR) to inhaled A. fumigatus 2 % in an orotracheally intubated allergic mouse. Decreases in GL, Cdyn, and EF_50 _values were associated with small declines in VT, f and TE. The ordinate at the bottom indicates the photometric signal of the allergen aerosol challenge.

The allergen-specific early airway response (EAR) to A. fumigatus was investigated in allergic mice on day 21 (Fig. 1 and 2). To avoid unbalanced challenges with allergen or saline, each group was separated into two subgroups for invasive and noninvasive measurement of pulmonary function.

Invasive recordings of EAR in allergic mice showed significant decreases in simultaneously measured GL, Cdyn, and EF_50 _compared with controls thus indicating an allergen-specific EAR to A. fumigatus. As shown in Figure 1, the most prominent alteration was shown for GL with a reduction by -62.1 ± 5.1 % (P < 0.001 vs. control) compared with a reduction by -48.8 ± 8.3 % in Cdyn (P < 0.001 vs. control), and a decrease by -34.5 ± 5.1 % in EF_50 _(P < 0.001 vs. control). The bronchoconstrictive response started within 7 ± 4 minutes (mean ± SD) after start of exposure and reached its maximum within 14 ± 3 min (mean ± SD). Figure 2 illustrates a characteristic time-response course of the EAR in an anesthetized, orotracheally intubated allergic mouse.

To determine if decreases in invasively monitored EF_50_, relate to changes in GL and Cdyn, we analyzed the agreement between these measurements by the method of Bland and Altman. Although all three parameters, Cdyn, GL and EF_50_, adequately reflected the pronounced EAR in allergic mice there was enhanced variation between GL vs. EF_50_, GL vs. Cdyn and EF_50 _vs. Cdyn in response to specific allergen challenge. As shown in Table 2, EF_50 _tended to underestimate the decreases in GL by -27.6 %, and by -14.3 % for Cdyn in allergic animals. In contrast, a very good agreement between EF_50_, GL and Cdyn values was found for control mice, with mean differences ranging from -2.4 to -6.1 %.

**Table 2:** Bland-Altman analysis of the differences in GL, EF_50 _and Cdyn.

		Early AR		Cholinergic AR	
Group	Parameters	Mean ± SD (95% CI)	Upper limit (95% CI) Lower limit (95% CI)	Mean ± SD (95% CI)	Upper limit (95% CI) Lower limit (95% CI)

Allergic	EF_50 _vs. GL	-27.6 ± 17.8 (-42.6/-12.7)	8.0 (-17.8/33.9) -63.3 (-89.2/-37.5)	-0.7 ± 0.7 (-1.3/0.1)	0.7 (-0.3/1.8) -2.1 (-3.2/-1.1)
	GL vs. Cdyn	13.3 ± 21.9 (-5/31.7)	57.1 (25.4/88.8) -30.5 (-62.2/1.2)	0 ± 0.2 (-0.2/0.2)	0.4 (0.1/0.7) -0.4 (-0.7/-0.1)
	EF_50 _vs. Cdyn	-14.3 ± 29.5 (-39/10.3)	44.7 (2/87.4) -73.3 (-116/-30.6)	-0.7 ± 0.9 (-1.4/0)	1 (-0.2/2.3) -2.5 (-3.7/-1.2)

Control	EF_50 _vs. GL	-2.4 ± 9.5 (-10.4/5.5)	16.6 (2.8/30.5) -21.5 (-35.3/-7.7)	-2.9 ± 3.3 (-5.7/-0.2)	3.7 (-1.1/8.5) -9.5 (-14.3/-4.7)
	GL vs. Cdyn	-3.7 ± 10.4 (-12.2/5.1)	17.2 (2.1 to 32.3) -24.6 (-39.7/-9.4)	1.4 ± 1.8 (-0.2/2.9)	5 (2.4/7.7) -2.3 (-4.9/0.4)
	EF_50 _vs. Cdyn	-6.1 ± 9.1 (-13.8/-1.5)	12.2 (-1.1/25.4) -24.4 (-37.6/-11.2)	-1.5 ± 3.5 (-4.5/1.4)	5.5 (0.4/10.7) -8.6 (-13.8/-3.5)

Differences in simultaneous invasive measurements of GL, EF_50 _and Cdyn for allergic and control mice during EAR and cholinergic AR. Values are means ± SD (95 % confidence intervals (CI) in brackets) for 8 animals per group. The upper and lower limits of agreement (means ± 2 SD) as well as the corresponding 95 % CI intervals (in brackets) are shown. Values for the EAR represent the % change from baseline, whereas the values for cholinergic AR show the absolute PD50 values in μg MCh.

Noninvasive measurements of pulmonary function in allergic mice also demonstrated a marked allergen-specific EAR as manifested by a significant decline by -44.6 ± 6.2 % in EF_50 _compared with that in control animals (P = 0.002, Fig. 1). The magnitude of the response was similar to the decline observed with invasively recorded EF_50_. Reduced EF_50_values were accompanied by decreased VT values and – in contrast to invasive measurements – by decreased f and increased TE values.

### Invasive vs. noninvasive determination of cholinergic AHR

To further characterize the utility of noninvasive vs. noninvasive pulmonary function tests, AR to increasing doses of aerosolized MCh, was investigated 48 h after EAR recordings in the same animals. Baseline GL, Cdyn and EF_50 _values were not significantly different from initial baseline values.

**Figure 3:**
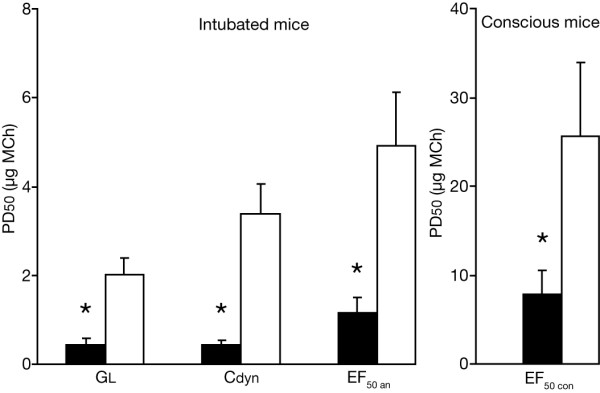
**Cholinergic AR. **Magnitudes of cholinergic AHR, 48 h after EAR, expressed as PD50 values, which is the dose of MCh required to reduce either GL, Cdyn or EF_50 _to 50 % of their respective baseline values) of invasively measured GL, Cdyn and EF_50 _(A) as well as of noninvasively recorded EF_50 _(B). Allergic mice (black columns) showed significantly lower PD50 values compared with controls (white columns). Baseline values were not significantly different from initial baseline values 48 h before and were within the means ± SD as listed in Table 1. Values are means ± SE, n = 8 per group, *p < 0.05 vs. control.

MCh exposure elicited a dose-related reduction in GL, Cdyn, and EF_50 _values in the intubated animals that was significantly enhanced in allergic mice (p < 0.05 vs. control group). The magnitude of cholinergic AR was significantly higher for GL and Cdyn compared with simultaneously measured EF_50 _(P = 0.027). Accordingly, the mean PD50 causing a decrease in Cdyn, EF_50 _and GL to 50 % baseline was 0.4 ± 0.1 for GL, 0.4 ± 0.1 for Cdyn, and 1.2 ± 0.4 μg MCh for EF_50 _in allergic mice (Fig. 3). The respective mean PD50 values for control animals were significantly higher: 2 ± 0.4 for GL (P = 0.001), 3.4 ± 0.7 for Cdyn (P = 0.002), and 4.9 ± 1.2 μg MCh for EF_50 _(P = 0.008). The dose-related decreases in EF_50 _were accompanied by increases in esophageal pressures. At the level of the 50% decline in EF_50 _(PD50), the peak esophageal pressure increased 121 ± 13 % for the allergic mice and 104 ± 16 % for the control group.

**Figure 4:**
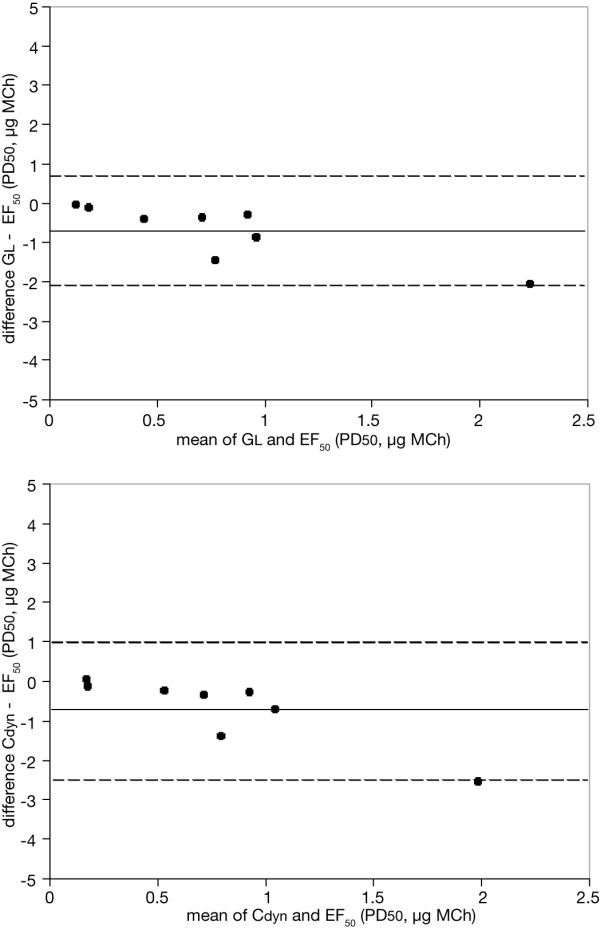
**Bland-Altman plots. **Individual differences in the degree of MCh-induced bronchoconstriction between invasively measured EF_50 _and GL and between EF_50 _and Cdyn, are plotted against the average corresponding values (expressed as PD50, μg MCh). The solid line represents the mean of the differences, the dashed lines show the upper and lower limits of agreement.

The peak responses for GL, Cdyn and EF_50 _occurred within 1 min after challenge and recovered to within 10–20 % of the baseline before MCh exposure during 1–3 min. Agreements between Cdyn, EF_50 _and GL were excellent, the mean ranging from 0 to -0.71 μg MCh for the allergic group and from -2.9 to 1.38 μg MCh for the control group (Table 2). Figure 4 shows the corresponding Bland-Altman plots of the differences between EF_50 _vs. GL and between EF_50 _vs. Cdyn against the mean of both values in allergic animals.

Noninvasive determination of EF_50 _also showed that allergic mice were significantly more responsive to MCh, as indicated by significantly lower PD50 values for EF_50 _when compared with controls (P = 0.032) (Fig. 3).

### Allergic airway inflammation

The A. fumigatus-sensitized and boosted animals showed significant increases in eosinophils and lymphocytes in BAL fluid (Table 3) compared with control mice. This indicates the presence of an inflammatory response in the lungs of allergic mice. The intubated animals receiving aerosols directly via the orotracheal tube had slightly higher numbers of leukocyte populations compared with conscious mice (statistically not significant).

**Table 3:** Cellular composition of BAL fluid

	Control mice conscious	Allergic mice conscious	Control mice anesthetized	Allergic mice anesthetized
Eosinophils, × 10^4^	< 1	7.9 ± 5.6*	< 1	13.4 ± 9.3*
Lymphocytes, × 10^4^	< 1	3.2 ± 2.2*	0.5 ± 0.4	1.8 ± 1.6*
Neutrophils, × 10^4^	< 1	1.3 ± 1	1.9 ± 1.3	2.7 ± 4.1
Macrophages, × 10^4^	12.3 ± 3.3	13.7 ± 3.1	22 ± 9.2	16.7 ± 6.1

Values are means ± SD from 8 animals per group. Eosinophils and lymphocytes recovered from bronchoalveolar lavage (BAL) fluid 48 hours after allergen challenge were increased in both conscious and intubated allergic mice. *P < 0.05 vs. control mice.

### Impact of hyperoxia on EF_50 _measurements in C57BL/6 mice

To examine how EF_50 _correlates with direct lung resistance measurements, C57BL/6 mice were exposed to 100% oxygen for 48 h. Table 4 lists the hyperoxia-induced changes detected by invasive and noninvasive lung function measurements compared with control animals. Noninvasive recordings revealed no significant differences in breathing rate, TE, VT, and EF_50 _between control and hyperoxia mice after 48 h of hyperoxia. Likewise, direct measurements of pulmonary mechanics in the same animals did not show any differences in EF_50_, Cdyn and RL values, thus confirming the absence of airway constriction in both groups.

**Table 4:** Impact of hyperoxia over 48 h on invasively and noninvasively measured respiratory parameters

Noninvasive measurement						Invasive measurement						
	EF_50_	TE	VT	f		RL	Cdyn	GL	EF_50_	TE	VT*	f
Control	2.36 ± 0.12	0.13 ± 0.01	0.20 ± 0.01	251 ± 14		1.44 ± 0.27	0.017 ± 0.004	0.72 ± 0.15	1.01 ± 0.13	0.3 ± 0.03	0.11 ± 0.02	106 ± 9
Hyperoxia	2.30 ± 0.41	0.14 ± 0.02	0.20 ± 0.02	245 ± 41		1.27 ± 0.29	0.018 ± 0.007	0.85 ± 0.18	0.93 ± 0.15	0.32 ± 0.04	0.14 ± 0.02	99 ± 15

Values are means ± SD from 8 C57BL/6 mice per group. *P < 0.05 vs. control mice. VT: tidal volume, EF_50_: tidal midexpiratory flow, TE: time of expiration, f: respiratory rate, RL: pulmonary resistance, Cdyn: dynamic compliance, GL: pulmonary conductance (GL = 1/RL).

## Discussion

In the present study we have evaluated the sensitivity and reliability of repeatable noninvasive versus invasive pulmonary function tests to sequentially measure AR in response to specific allergen and cholinergic challenge in spontaneously breathing mice. Our results demonstrate that both systems reflect the allergen-specific early AR and cholinergic AHR of allergic compared with control mice.

The ability to manipulate the mouse genome has opened up new opportunities to develop mouse models of allergic asthma that demonstrate spontaneous or chronic disease [24]. For a proper phenotyping of AR in experimental models it is crucial to monitor pulmonary function as reliably as possible. One way to achieve this is a novel in-vivo method that combines repetitive recordings of classical pulmonary mechanics with cholinergic aerosol challenges in orotracheally intubated mice [4]. Despite being an accurate measurement of classical pulmonary function on multiple occasions, this invasive method does not readily allow for rapid screening of pulmonary function in large numbers of animals.

In contrast, noninvasive head-out body plethysmography has been shown to yield stable and reliable on-line measurements of AR in several conscious mice at a time and serves as a suitable and valid tool to complement the traditional measures of pulmonary mechanics [13,14,16,22,25]. Limitations of previous EF_50 _validation studies in mice particularly have included pleural catheterization with the inability to conduct reproducible measurements, the contribution of upper airway resistance and intravenous rather than aerosol challenge [14,17]. These methodological shortcomings introduced variability into the results which made them difficult to compare with other invasive techniques [10].

The current report intended to overcome such problems in that GL, Cdyn and EF_50 _were measured simultaneously in intact mice including local aerosol challenges via an orotracheal tube. In parallel, noninvasive determinations of EF_50 _were performed in allergic and control mice. The noninvasive experiments relied on methodologies identical to those used in our previous mice studies to facilitate comparisons [14,17,22].

The values for respiratory parameters measured from both conscious and anesthetized BALB/c mice were reproducible and comparable with those reported previously for this strain (Table 1) [4,14,26]. The changes in respiratory patterns observed in anesthetized mice were associated with increased expiratory time, decreased f, and decreased EF_50 _values, events likely related to anesthetic effects on neural respiratory control. The independence of EF_50 _recordings from changes in frequency has been demonstrated in previous investigations [14,15].

To examine the sensitivity of noninvasive and invasive indices of bronchoconstriction, we monitored allergen-specific EAR and, 48 h later, performed MCh dose-response studies in the same allergic animals compared with controls. Challenge with aerosolized A. fumigatus resulted in significant reductions in Cdyn, GL and in EF_50 _values in allergic mice compared with (sham-exposed) control animals. Demonstration of allergen-specific EAR in allergic mice was followed by cholinergic AHR that was linked with a pronounced influx of neutrophils and eosinophils in BAL fluid. Consistent with previous results, invasively recorded EF_50 _was slightly less sensitive in detecting the maximum degree of bronchoconstriction to A. fumigatus and MCh compared with GL and Cdyn recordings [15].

Agreement between invasively measured EF_50_, GL and Cdyn during EAR and cholinergic AHR was good, although there was increased variability at the time of EAR in allergic mice (Table 2). This variability may reflect different sensitivities of GL, EF_50 _and Cdyn to the airway and tissue components of total pulmonary resistance [3,16]. Related to this issue, is a previous study indicating that mice with airway inflammation experience quite heterogeneous airway narrowing and airway closure during airway smooth muscle contraction [27].

Nevertheless, despite this variability, it is important to emphasize that the noninvasive measurement of EF_50 _still reflected the enhanced AR to A. fumigatus and MCh in allergic relative to control mice (Figs. 1, 3). Thus, although the calculated inhalation doses for A. fumigatus and MCh in conscious mice may be not as accurate as in intubated mice, the observed EF_50 _responses still reflect airway constriction. These findings indicate that EF_50 _can distinguish between different magnitudes of AR and reflects the changes with GL and Cdyn during bronchoconstriction at least under the conditions of this study. Moreover, the relation of the cholinergic EF_50 _response between allergic and control animals was similar for invasive and noninvasive measurements (Figure 3). The higher PD50 values for EF_50 _in conscious compared with intubated animals to MCh challenge can be explained by methodological issues. Administration of aerosols directly into the lungs via an orotracheal tube results in aerosol deposition mainly in the parenchyma. In conscious animals there will be substantial deposition in the nasal passages and upper airway, which should lead to the higher PD50 values observed. The AR, as measured noninvasively by EF_50_, may also be partly affected by altered upper airway resistance. However, because of the rapid onset and resolution of the response, it seems unlikely that edema or mucus hypersecretion in these upper airways was responsible for the increased AR.

In agreement with other investigations, decreases in EF_50_, as measured by noninvasive head-out body plethysmography, were linked with decreased frequency and VT values and increasing values for TE [12,14,15]. In contrast, no relevant impact on frequency and TE was found in anesthetized, intubated mice during bronchoconstriction.

Concerns with noninvasive EF_50 _recordings include the uncertainty about the exact degree and localization of bronchoconstriction as well as the potential contribution of upper airway resistance. Due to methodological differences, comparisons between invasive and noninvasive measures are of indirect, qualitative nature. A quantitative comparison, however, is directly available from the intraindividual differences between simultaneously measured EF_50 _and GL in unconscious mice. Because EF_50 _tends to underestimate the magnitude of bronchoconstriction (discussed below) it is still unclear whether this limits its use in detecting less marked changes in airway hyperresponsiveness than those induced in high-reponder models. As a result, EF_50 _measures should be confirmed with direct assessments of pulmonary resistance under these circumstances. Despite these methodological restrictions, the observed EF_50 _responses still reflected the enhanced AR to ACh and allergen under the conditions of this study.

In comparison with the widely used Penh method, EF_50 _differs substantially in several important ways: EF_50 _decreases with bronchoconstriction and in line with invasively measured lung resistance or conductance is linked with a decline in VT during bronchoconstriction [7,28]. Even more importantly, EF_50 _has a physical meaning (ml/s), allows direct comparison from one animal to another and is closely related to airway resistance. Indeed, if it were possible to know the esophageal pressure in the conscious animals, one could calculate a precise lung resistance. If we assume that esophageal pressure does not change, then changes in the EF_50 _would be directly proportional to the lung resistance. However, in the anesthetized animals, we found that the esophageal pressure actually increased as the airways constricted, perhaps in response to the increased resistance and lower air flow. This suggests that the EF_50 _in conscious animals may underestimate the actual changes in lung resistance. Despite this quantitative limitation, the method seems far more representative of changes in resistance than other noninvasive methods, and the approach allows for direct quantitative comparisons from animal to animal. The commonly measured Penh has no theoretical linkage to lung resistance, and its usefulness was further weakened by recent reports, one of which showed that changes in Penh were no better than simply measuring TE to assess AR in common strains of laboratory mice [6]. It is also known that a decline in noninvasively measured EF_50 _is associated with an increase in TE [12,14]. However, it is important to note that conditions entirely unrelated to bronchoconstriction, such as sensory irritation, will also result in increasing TE values [12,29].

Another report demonstrated that Penh was inadequate for characterization of pulmonary mechanics in the context of hyperoxia-induced changes in C57BL/6 mice [18]. These authors pointed out that Penh may significantly overestimate the actual changes in lung resistance after 24 and 48 h of hyperoxia. Interestingly, increases in Penh were accompanied by decreased TE and rising VT and f. This contrasts with the above-mentioned observation of decreased VT during bronchoconstriction as observed with EF_50 _and invasive pulmonary function methods [4,28]. Our study in C57BL/6 mice showed a consistent relationship between EF_50 _and lung resistance measurements in reponse to 48 h hyperoxia, thus indicating non-constricted airways. These data support the concept that EF_50 _more reliably reflects airway resistance than Penh, which is largely a function of respiratory timing.

## Conclusion

In conclusion, this study investigated the utility of repetitive invasive vs. noninvasive techniques to determine AR to allergen and cholinergic challenge in intact, spontaneously breathing mice. We demonstrated allergen-specific EAR to A. fumigatus followed by cholinergic AHR in allergic mice compared with controls. Our results show that the noninvasive EF_50 _method is directly related to lung resistance, and is thus particularly appropriate for quick and repeatable phenotyping of airway function in large numbers of conscious mice.

## Competing interests

The author(s) declare that they have no competing interests.

## Authors' contributions

TG participated in the design and coordination of the study and drafted the manuscript. MZ and RB carried out the lung function experiments. RK participated in the data analysis of all experiments, AB carried out the cytological and ELISA tests. WM helped to draft the manuscript. JMH and NK participated in the coordination and analysis of the study. HGH conceived of the study, and participated in its design and analysis. All authors read and approved the final manuscript
